# Arterial pulsation artifact mimicking spiked helmet sign: a case report

**DOI:** 10.3389/fcvm.2026.1648793

**Published:** 2026-03-12

**Authors:** Huaisheng Ding, Chengyu Wang, Jingyu Kan, Jingwen Ding, Luo Yao, Peng Li

**Affiliations:** 1 Department of Cardiology, West China Hospital Sichuan University, Meishan Hospital, Meishan, China; 2 Department of Cardiology, Meishan People's Hospital, Meishan, China

**Keywords:** arterial pulsation, case report, diagnostic error, electrocardiogram artifact, limb lead electrocardiography, spiked helmet sign

## Abstract

This case report presents an 86-year-old female patient admitted for the surgical evaluation of a mediastinal cystic mass. Initial electrocardiogram (ECG) showed ST-segment and T-wave changes resembling the spiked helmet sign. Considering the patient's stable vital signs and the absence of ST-segment changes in lead I, the possibility of ECG interference was suspected. Further investigation and repeated ECG, with adjustments to electrode placement away from the left lower limb artery, confirmed that the changes were due to arterial pulsation interference rather than a true cardiac abnormality. This case highlights the critical need to differentiate true cardiac ischemia from artifact-mediated ECG changes, particularly in hemodynamically stable patients.

## Introduction

The spiked helmet sign (SHS) is an ominous electrocardiographic (ECG) marker characterized by a distinctive triad of J-point elevation, convex ST-segment changes, and macroscopic T-wave alternans. First described by Littmann et al. in 2011, SHS is typically associated with critical illness, sympathetic hyperactivity, and high mortality risk due to its link with malignant ventricular arrhythmias (1, 2).While SHS commonly arises from pathological conditions such as intracranial hemorrhage, sepsis, or myocardial repolarization disorders (3–5), it is crucial to recognize that similar ECG patterns may result from extracardiac artifacts. Physiological interference—including somatic tremor, patient movement, or arterial pulsation—can mimic pathological ST-T changes, leading to potential misdiagnosis and unnecessary interventions (6–9). This case underscores the imperative to differentiate true electrical instability from artifact-mediated pseudosigns, especially when ECG abnormalities are inconsistent with the patient's clinical condition. Herein, we present an instance of SHS-mimicking changes solely induced by left lower limb arterial pulsation interference, which was initially mistaken for an effect of a mediastinal mass. The resolution of ECG abnormalities after electrode repositioning—away from the arterial pulsation source—demonstrates the critical role of technical adjustments guided by Einthoven's lead theory (10).

## Case report

An 86-year-old female patient was admitted to our hospital for the preoperative evaluation of a mediastinal cystic mass. Chest computed tomography (CT) revealed a large cystic mass in the posterior-inferior mediastinum, measuring approximately 56 mm × 55 mm × 92 mm, located posterior to the heart and anterior to the esophagus. A transthoracic echocardiogram was normal, showing no structural or functional abnormalities. The patient's vital signs at the time of the initial ECG were stable: blood pressure was 138/75 mmHg, heart rate was 65 beats per minute, respiratory rate was 16 breaths/min, and oxygen saturation was 98% on room air. She denied any chest pain, palpitations, or shortness of breath. Laboratory returned with a cardiac troponin I level of 0.02 ng/mL (normal range <0.05 ng/mL), which ruled out acute myocardial injury. Serum electrolyte levels, including potassium (4.2 mmol/L), magnesium (0.85 mmol/L), and calcium (2.25 mmol/L), were all within normal limits. The patient has not taken any medication recently.

**Figure 1:**
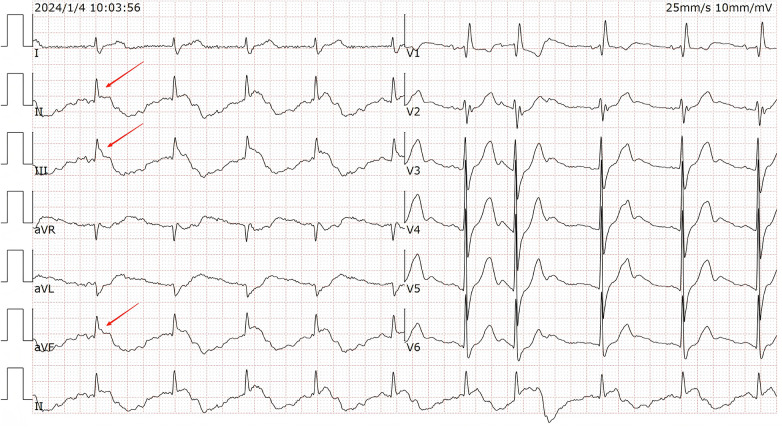
The initial ECG showed Sinus rhythm with RBBB, a sharp R wave followed by convex ST-segment changes, and marked QT prolongation. Red arrows pointing specifically to the “spiked” R waves and convex ST-segment elevations in leads II, III, and aVF. Lead I QT interval: 442 ms,lead II QT interval: 723 ms.

A 12-lead electrocardiogram (ECG) was performed using a digital multi-channel electrocardiograph (iMAC 100, Zoncare Biomedical Electronics Co., Shenzhen, China; filter setting: 0.05–150 Hz) when admitted, the initial ECG evaluation demonstrated ST-segment and T-wave changes and as shown in the Figure 1.

Specifically, the ECG showed a sinus rate of 65 beats per minute, complete right bundle branch block(RBBB), atrial premature beats, a sharp R wave followed by convex ST-segment changes, and marked QT prolongation, These changes were most prominent in the inferior leads (II, III, and aVF), as indicated by the red arrows in Figure 1. This ECG pattern resembled the SHS, first described by L Littmann in 2011 (1). Initially, we considered whether the changes might be caused by elevation of the mediastinum due to compression by the large mediastinal tumor. However, the patient remained completely asymptomatic with stable vital signs.

## Diagnostic reasoning

The striking ECG findings in an otherwise stable, asymptomatic patient prompted a systematic diagnostic approach. Two main possibilities were considered: (1) true pathological SHS, and (2) ECG artifact mimicking SHS.

Several observations favored artifact. First, the patient was hemodynamically stable and asymptomatic—inconsistent with true SHS, which is associated with critical illness (1, 2). Second, troponin and electrolytes were normal, excluding ischemia or metabolic causes. Third, lead I—which does not involve the left leg electrode—showed no abnormalities (QT 442 ms), while leads II, III, and aVF showed dramatic changes (QT 723 ms in lead II). According to Einthoven's triangle theory (10), this pattern localized the interference to the left lower limb.

The large mediastinal mass on imaging was a potential diagnostic distraction, but the clinical stability and lead I sparing pointed to a simpler explanation. Based on this reasoning, we hypothesized that the SHS-like pattern was caused by arterial pulsation at the left leg electrode. To test this hypothesis, we performed a simple, deliberate intervention.

## Intervention and outcome

Given the patient's persistent clinical stability and the suspicion of artifact, a cardiology consultation was obtained. Three days after admission, a repeat 12-lead ECG was performed under our supervision. Based on the initial hypothesis that arterial pulsation from the left lower limb was the source of interference, the left lower limb electrode was deliberately placed away from the palpable arterial pulsation site—on the anterolateral thigh. All other electrodes were placed in standard positions, and skin contact was optimized.

**Figure 2:**
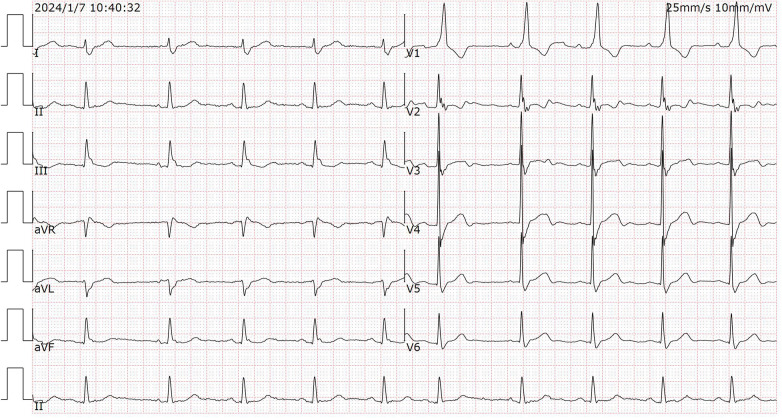
The repeat ECG showed sinus rhythm (SR) without obvious ST-T deviation.

The repeat tracing demonstrated complete normalization of the previously observed ST-T changes and QT interval (Figure 2). The fact that the ECG abnormalities resolved immediately upon electrode repositioning, in a patient who remained clinically stable throughout the three-day interval, confirmed that the initial findings were caused by an artifact rather than a transient ischemic event or structural compression. The lead involvement (II, III, aVF) pointed to the left leg as the source, with arterial pulsation being the most plausible cause.

## Timeline

Day 1 (Admission): Initial ECG shows SHS-like changes; artifact suspected based on clinical stability and lead I sparing. Patient observed without intervention.
Day 4: Repeat ECG performed after electrode repositioning shows complete normalization.
Day 6: Patient undergoes successful surgical resection of mediastinal cyst.
Day 10: Discharged home without complications.
6-month follow-up: Patient remains asymptomatic; repeat ECG shows no abnormalities.

## Follow-up and patient perspective

At the 6-month follow-up visit, the patient reported no cardiac symptoms and a repeat ECG was normal. She was informed about the initial ECG findings and the diagnostic process; she expressed relief that the abnormalities were not indicative of a heart problem and understood the importance of the electrode adjustment. She remained satisfied with her care and experienced no adverse events related to the diagnostic process.

## Discussion

SHS is a rare electrocardiographic marker initially described by Littmann et al. as an indicator of critical illness and high mortality risk (1). Its pathophysiological basis involves sympathetic hyperactivity, which promotes ventricular repolarization dispersion and QT-interval prolongation, creating a substrate for malignant ventricular arrhythmias including torsade de pointes (TdP) (3, 11). Mechanistically, SHS stems from imbalanced transmural repolarization gradients, triggered by genetic channelopathies, myocardial ischemia, or systemic stressors such as sepsis, intracranial hemorrhage, or pressure overload (2, 4, 5, 11–13). These mechanisms collectively establish SHS as an electrocardiographic harbinger of electrical instability in critically ill patients (1, 2).

Although ECG artifacts mimicking the spiked helmet sign have been previously reported in settings such as arm arteriovenous fistulas or abdominal aortic pulsation (7–9), the current case exhibits two distinctive features that enhance its educational value. First, it identifies arterial pulsation from the left lower limb as a specific and underrecognized source of artifact in inferior leads, thereby expanding the clinician's differential for such patterns. Second, and more notably, the concomitant presence of a large mediastinal mass introduced a significant diagnostic distraction, creating a compelling rationale to attribute the repolarization abnormalities to mechanical compression. This particular context elevates the case from a mere technical observation to a instructive example of cognitive bias, emphasizing that even highly plausible structural etiologies should not be considered until basic technical artifacts have been systematically excluded through a methodical approach.

However, SHS-like ECG patterns may also stem from non-pathological artifacts, necessitating careful differentiation to avoid misdiagnosis. ECG artifacts can be divided into physiological and non - physiological categories (6). Physiological sources may include somatic muscle tremor, excessive movement, and arterial pulsation. Arterial pulsation - related artifacts have been reported in cases such as arm arteriovenous fistula (7), atypical radial artery (8), and abdominal aortic pulsation (9).

According to the Einthoven triangle theory (10–15), lead I reflects the electrical difference between the right arm and left arm; lead II, between the right arm and left leg; and lead III, between the left arm and left leg. When artifacts originate from a particular limb, specific leads remain unaffected. The precise localization of the artifact to the left lower limb can be systematically understood by applying the principles of Einthoven's triangle, as detailed in Table 1. The standard limb leads measure the electrical potential difference between specific pairs of electrodes. As demonstrated in the table, an artifact originating from the left leg (LL) electrode site will be incorporated into the recordings of any lead that uses LL as an input. This explains why the spiked helmet sign-mimicking pattern was present in leads II, III, and aVF. Conversely, lead I, which is derived solely from the RA and LA electrodes, remains unaffected, providing the crucial diagnostic clue that isolated the source of interference to the left lower limb.

**Table 1:** Localization of electrocardiographic artifact using Einthoven's triangle theory: a case example of left lower limb interference.

Lead	Involved electrodes (input)	Mathematical formula	Influence of artifacts in the LL area	In this case
I	RA to LA	I = RA - LA	Unaffected(LL electrode does not participate in this circuit)	Normal(No SHS)
II	RA to LL	II = RA - LL	Affected(Artifacts are added to the LL input terminal)	Abnormal(SHS)
III	LA to LL	III = LA - LL	Affected(Artifacts are added to the LL input terminal)	Abnormal(SHS)
aVF	Augmented Vector Foot	aVF≈LL - (RA + LA)/2	Affected(This lead heavily relies on LL potential)	Abnormal(SHS)

RA, right arm; LA, left arm; LL, left leg.

This case serves as a reminder to clinicians that ECG findings should be interpreted in the context of the patient's overall clinical presentation. When ECG abnormalities do not correlate with the patient's clinical status, a comprehensive evaluation for potential extracardiac causes is crucial. This may involve repeated ECGs, imaging studies, or other diagnostic tests to confirm or exclude suspected extracardiac interference.

## Limitations

While this case provides valuable insights, it has several limitations. First, as a single case report, the findings may not be generalizable to all patients with SHS-like ECG changes. Second, although the intervention strongly supports arterial pulsation as the cause, we did not use simultaneous arterial line monitoring or mechanical vibration detection to definitively confirm the artifact source. Third, we cannot completely exclude the possibility that other factors, such as subtle changes in electrode contact or patient positioning, contributed to the ECG normalization. Finally, long-term outcomes beyond 6 months are not available.

This case serves as a reminder that when ECG abnormalities do not correlate with a patient's clinical stability, a systematic evaluation for extracardiac causes—including repeated ECGs with technical adjustments—should be undertaken before attributing findings to structural or ischemic pathology.

## Conclusion

In conclusion, this case highlights the importance of careful interpretation of ECG changes and the need to consider extracardiac factors, especially arterial pulsation interference, while evaluating ECGs. Clinicians should remain vigilant to avoid misdiagnosis and ensure accurate management decisions.

## Data Availability

The original contributions presented in the study are included in the article/Supplementary Material, further inquiries can be directed to the corresponding author.
